# An array of basic residues is essential for the nucleolytic activity of the PHP domain of bacterial/archaeal PolX DNA polymerases

**DOI:** 10.1038/s41598-019-46349-8

**Published:** 2019-07-09

**Authors:** Guillermo Rodríguez, María Teresa Martín, Miguel de Vega

**Affiliations:** 1 Centro de Biología Molecular “Severo Ochoa” (Consejo Superior de Investigaciones Científicas-Universidad Autónoma de Madrid), Nicolás Cabrera 1, 28049 Madrid, Spain; 2 Centro Nacional de Biotecnología (Consejo Superior de Investigaciones Científicas), Darwin 3, 28049 Madrid, Spain

**Keywords:** DNA damage and repair, Enzymes, DNA damage and repair, Enzymes

## Abstract

Bacterial/archaeal family X DNA polymerases (PolXs) have a C-terminal PHP domain with an active site formed by nine histidines and aspartates that catalyzes 3′-5′ exonuclease, AP-endonuclease, 3′-phosphodiesterase and 3′-phosphatase activities. Multiple sequence alignments have allowed us to identify additional highly conserved residues along the PHP domain of bacterial/archaeal PolXs that form an electropositive path to the catalytic site and whose potential role in the nucleolytic activities had not been established. Here, site directed mutagenesis at the corresponding *Bacillus subtilis* PolX (PolXBs) residues, Arg^469^, Arg^474^, Asn^498^, Arg^503^ and Lys^545^, as well as to the highly conserved residue Phe^440^ gave rise to enzymes severely affected in all the nucleolytic activities of the enzyme while conserving a wild-type gap-filling activity, indicating a function of those residues in DNA binding at the PHP domain. Altogether, the results obtained with the mutant proteins, the spatial arrangement of those DNA binding residues, the intermolecular transference of the 3′-terminus between the PHP and polymerization active sites, and the available 3D structures of bacterial PolXs led us to propose the requirement to a great degree of a functional/structural flexibility to coordinate the synthetic and degradative activities in these enzymes.

## Introduction

Genome stability maintenance is critical to all forms of life. Therefore, the enormous variety of DNA damages has imposed the evolution of specific DNA repair pathways where a plethora of specific enzymatic activities repair those lesions that otherwise could cause a blockage of essential biological processes as genome replication and transcription^1^.

Among the DNA repair pathways, base excision repair (BER) and nonhomologous end joining (NHEJ) stand out. On the one hand, BER is the most frequently used DNA repair pathway *in vivo*. It has been estimated that it repairs more than 20,000 DNA lesions per cell per day as it is responsible for mending the broad spectrum of non-bulky and non-helix distorting lesions caused by reactive oxygen species and alkylating agents^2^. Although a multibranched pathway^3^, the general BER process starts with the release of the lesion by a DNA glycosylase. The 5′ side of the resultant AP site is cleaved by an AP endonuclease, giving rise to a gapped molecule further filled by a DNA polymerase. On the other hand, NHEJ is one of the pathways responsible for mending DNA double-strand breaks (DSBs) that can be caused by irradiation and chemical agents, as well as they can arise during DNA replication^1^. Briefly, NHEJ starts with the binding to the DNA ends of the ring-shaped Ku70**/**80 heterodimer that further recruits the DNA-dependent protein kinase catalytic subunit that bridges the DNA ends. Such termini are usually damaged and have to be processed by nucleases as Artemis, APLF, and the MRN complex, or phosphatases as PNPK, giving rise to short gaps further filled by DNA polymerases.

The two repair pathways described above have a common gap-filling step carried out by a specialized DNA polymerase belonging to family X (PolXs)^4–13^. These enzymes are highly conserved in all the kingdoms of life^14^ and share a common structural organization that allow them to accommodate in short gaps to accomplish their efficient filling. Thus, PolXs share the general structure of mammalian Polβ, the first PolX described^15^. Such a structure consists in a C-terminal polymerization domain that comprises the universal fingers, palm and thumb subdomains, and is responsible for the addition of dNMPs to the 3′ end of the growing DNA chain. This domain is fused to an N-terminal 8-kDa domain that establishes interactions with the downstream strand of the gap. Whereas in the case of the eukaryotic Polλ, Polμ, terminal deoxyribonucleotide transferase (TdT) and yeast Pol4, the Polβ-like *core* is linked to an additional N-terminal BRCT domain, critical to interactions with NHEJ and V(D)J recombination factors^5,8,9,11,12,16^, in bacterial/archaeal PolXs is fused to a C-terminal Polymerase Histidinol Phosphatase (PHP) domain^17,18^. In the latter case, extensive biochemical analysis carried out in the PolX from the bacteria *Bacillus subtilis* (PolXBs) and *Thermus thermophilus* (ttPolX) have shown that the C-terminal PHP domain contains a nine-residue active site that binds divalent cations to catalyze nucleolytic activities^19–23^. Thus, these PHP domains have an apurinic/apyrimidinic (AP) endonuclease activity that in coordination with the polymerization one enables the PolX to recognize and incise at an AP site, further restoring the original non-damaged nucleotide; and Mn^2+^-dependent 3′-5′ exonuclease, 3′-phosphodiesterase and 3′-phosphatase activities that capacitate the polymerase to handle damage-containing 3′ termini^19,21–23^.

Here, we describe the role of highly conserved residues at the PHP domain of bacterial/archaeal PolXs. Thus, the biochemical characterization of PolXBs mutants at residues F440A, R469A, R474A, N498A, R503A, K545A, as well as of the deletion mutant Δ325-326 lead us to propose a role for these residues in DNA binding to allow proper function of the nucleolytic activities of the enzyme, AP-endonuclease, 3′-5′ exonuclease, 3′-phosphodiesterase and 3′-phosphatase. In addition, we have shown how the 3′-end generated after the action of the AP-endonuclease activity is transferred intermolecularly to the polymerization active site of PolXBs to allow further gap-filling during an *in vitro* BER reaction. Considering both, the results shown here and the available structures of ttPolX ternary complexes^24^ and of the apo PolX from *Deinococcus radiodurans* (PolXDr) we discuss the functional flexibility required to coordinate the synthetic and degradative activities of these enzymes.

## Results

### Site-directed mutagenesis in the PHP domain of PolXBs

**Figure 1:**
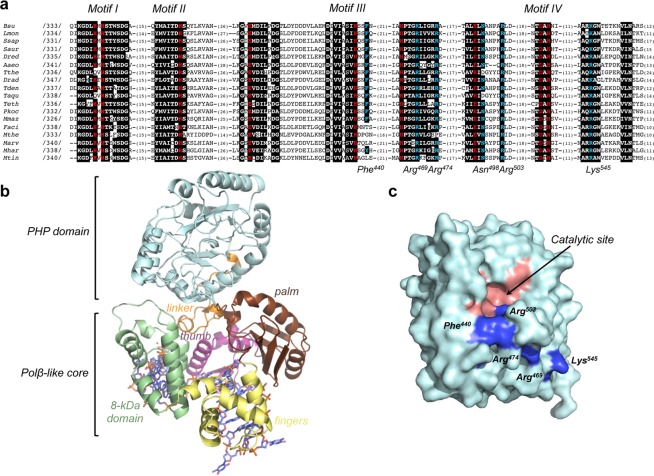
(**a**) Conserved regions of the C-terminal PHP domain of bacteria/archaeal PolXs. Numbers between slashes indicate the first aligned amino acid residue in each PolX. Due to the increasing number of sequences, only selected representative PolXs from the Eubacteria and Archaea domains are aligned. The abbreviations of the organisms and the accession numbers of the corresponding PolX are compiled in^36^, except for Teth, *Thermoanaerobacter ethanolicus* (EGD52315.1); Pkoc, *Planococcus kocurii* (AL579032.1); Marv, *Methanocella arvoryzae* (CAJ36494.1); Mhar, *Methanosaeta harudinacea* (AET65101.1); and Mtin, *Methanococcus tindarius* (ETA68502.1). The catalytic histidine, glutamic and aspartic residues are shown in red letters. Other highly conserved residues are indicated with white letters. Residues studied here are in blue letters. Alignment was done with the Mutalin tool (http://bioinfo.genopole-toulouse.prd.fr/multalin/multalin.html) and further adjusted manually. **(b)** *Structural model of the PolXBs/DNA complex*. The server SWISS-MODEL^40–43^ provided the model for PolXBs^36^, using as template the ternary complex of ttPolX (PDB code 3AUO^24^). **(c)** *Molecular surface representation of the PHP domain*. The catalytic active site is colored in red. Residues studied here are in dark blue.

Previous multiple alignments of the C-terminal PHP domain of bacterial/archaeal PolXs allowed to identify four conserved core regions (motifs I-IV) containing highly conserved histidines and aspartates^17^ (see Fig. 1a). Those residues were predicted to participate in catalysis since the corresponding ones in the *E. coli* YcdX protein, an isolated 27-kDa molecular weight protein that belongs to the PHP superfamily^17^, were coordinating three metal ions^25^. Such predictions were further confirmed by site-directed mutagenesis studies carried out in the corresponding PolXBs residues His^339^ and His^341^ (motif I), His^371^ (motif II), Glu^410^, His^437^ (motif III), His^465^, and Asp^526^ and His^528^ (motif IV)^19,20,23^ (colored in red in Fig. 1a) and in the homolog histidines and aspartates of ttPolX^21,22^. Thus, substitutions of those residues impaired both, the phosphodiester (3′-5′ exonuclease, AP-endonuclease and 3′-phosphodiesterase activities) and phosphoester (3′-phosphatase activity) bond hydrolysis. In the tertiary structure of the PHP domain the above residues are arranged to form a solvent exposed catalytic active site that is located nearby the molecular surface of the domain (Fig. 1c). As it can be observed, there is not any evident ssDNA binding cleft in the PHP domain.

The alignment shown in Fig. 1a allows also to identify additional electropositive residues (in blue) highly conserved along the PHP domain of bacterial/archaeal PolXs, corresponding to PolXBs residues Arg^469^, Arg^474^, Asn^498^, Arg^503^ and Lys^545^, and arranged in the tertiary structure in a way that form an electropositive path to the catalytic site (see Fig. 1c). This observation strongly suggests a potential DNA binding role for those residues that could be essential for the nucleolytic activities of these PolXs. Additionally, there is a moderately conserved Phe/Tyr residue in the motif III (PolXBs Phe^440^), placed just in the edge of the catalytic site. This residue has been predicted to play a role equivalent to that of Tyr^72^ of *E. coli* Endo IV in the detection of AP-sites by flipping the sugar-phosphate backbone at the AP site, the aromatic group controlling the active site hydrophobic environment to allow catalysis^20^.

To ascertain the functional importance of the PHP residues described above in allowing the catalysis of the nucleolytic activities of bacterial/archaeal PolXs, the corresponding PolXBs residues Phe^440^, Arg^469^, Arg^474^, Asn^498^, Arg^503^ and Lys^545^ were changed into Ala by site-directed mutagenesis, obtaining the derivatives F440A, R469A, R474A, N498A, R503A and K545A that were overproduced and purified as described in Materials and Methods.

Additionally, the 3D structure of the ttPolX ternary complex showed that the PHP domain forms a right angle with the DNA bound to the Polβ-like *core* by virtue of a 30 amino acids long linker^24^ (see also Fig. 1b). To analyze the importance of the proper orientation between both, the Polβ-like *core* and the PHP domains, we have shortened the PolXBs linker by deleting residues Ser235 and Ile236 (mutant derivative Δ325-326; see Materials and Methods).

### Mutations introduced at the PHP residues impair the nucleolytic activities of PolXBs

**Figure 2:**
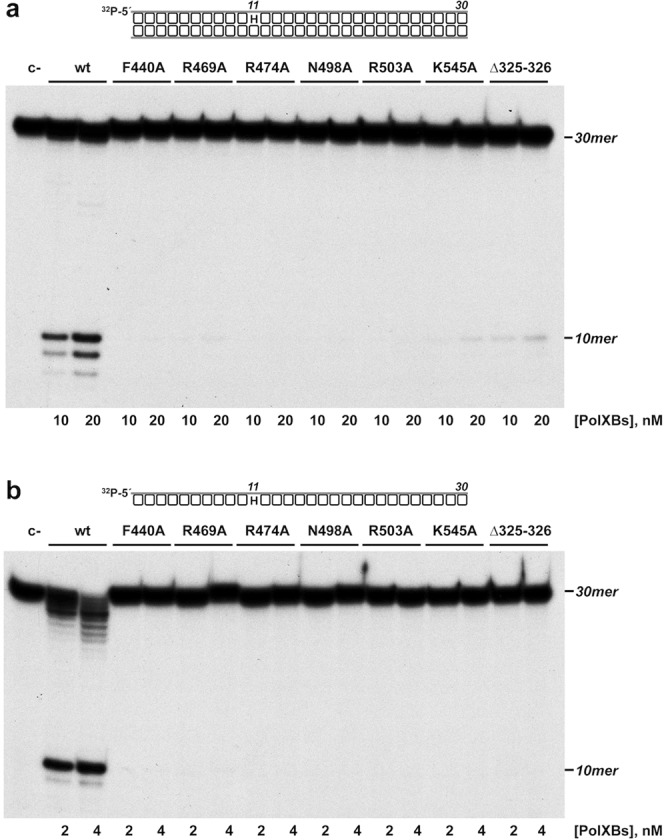
AP-endonuclease activity of the PolXBs mutants. The assays were carried out as described in Materials and Methods, incubating 4 nM of either the 30mer hybrid THF-11/THF-11C **(a)** or the ssDNA oligonucleotide THF-11 **(b)**, with the indicated concentration of either the wild-type or the specified PolXBs variant. Samples were incubated for either 5 min (**a**) or 2 min (**b**) at 30 °C. The products were resolved by denaturing PAGE and visualized by autoradiography. Full length gels are presented in Fig. S4.

**Figure 3:**
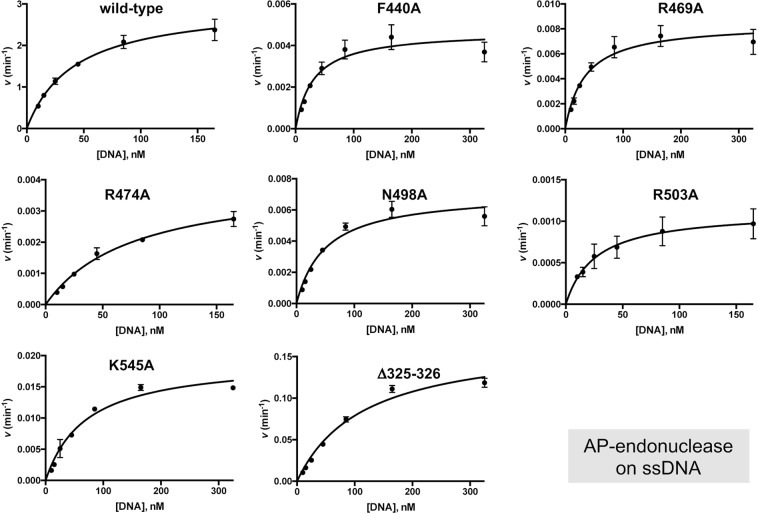
Steady-state analysis of the AP-endonuclease activity of the PolXBs derivatives. The assay was performed as described in Materials and Methods. Graphics show the turnover values (*v* in min^−1^) plotted as a function of ssDNA concentration (^32^P-5′ labeled oligonucleotide THF-11P; see Materials and Methods). The data were fitted to the Michaelis–Menten equation by least-squares nonlinear regression, and the resulting *kcat*, *Km* and catalytic efficiency (*kcat*/*Km*) values are given in Table 1.

As mentioned before, PolXBs is endowed with an AP-endonuclease activity that shares the catalytic site with the 3′-5′ exonuclease, being both activities governed by the same metal ligands located at the PHP domain^20^. This activity allows PolXBs to recognize and incise at AP sites, further restoring the original nucleotide by the polymerization activity, a result that led to propose the participation of PolXBs in the BER pathway^20,26^. To study the involvement of the PHP residues Arg^474^, Phe^440^, Arg^469^, Asn^498^, Arg^503^ and Lys^545^ in supporting the endonucleolytic reaction, PolXBs variants F440A, R469A, R474A, N498A, R503A and K545A were incubated in the presence of a DNA containing an internal tetrahydrofuran (THF; a stable analogue that mimics an AP site) at the 11 position (see Materials and Methods) and in the presence of 40 µM Mn^2+^, the optimal concentration for this activity (see Supplementary Fig. S1a). As shown in Fig. 2a, the wild-type polymerase hydrolyzed the phosphodiester bond at the 5′ side of the THF, giving rise to the expected 10mer reaction product, as previously described^20^. The shorter products are produced by the action of the 3′-5′ exonuclease activity on the 3′-end that results after incision at the AP site. As it can be observed, the AP endonuclease activity of PolXBs was severely impaired in all the mutant proteins. As the AP-endonuclease of PolXBs has been shown to be much more efficient on ssDNA substrates than on dsDNA^20^ we tested the ability of the PolXBs mutants to hydrolyze a THF-containing ssDNA. As shown in Fig. 2b, the protein variants exhibited a defective AP-endonuclease activity also on this substrate. To determine the kinetic parameters affected in the PolXBs mutants we performed AP-endonuclease assays under steady-state conditions (see Materials and Methods). As observed in Fig. 3 and Table 1, the catalytic efficiency (*k*_*cat*_/*K*_*m*_) exhibited by mutants F440A, R469A, R474A, N498A, R503A, K545A and Δ325-326 was 480-, 300-, 1384-, 514-, 1674-, 257- and 48-fold lower than that of the wild-type enzyme, respectively and primarily due to a very reduced catalytic rate. Interestingly, and as it can be observed in Fig. 3, although at a low extent, the PolXBs variants F440A, R469A, R474A, N498A, K545A, and Δ325-326 seem to exhibit a sigmoidal behavior that could be pointing to a some degree of cooperativity in the interaction of the polymerase with the DNA substrate. This fact led us to calculate the Hill coefficient from the steady-state data, as it is frequently considered an indicative measure of binding cooperativity^27^. As shown in Supplementary Table S1, except for mutant R503A, in the interaction with the DNA substrate of the wild-type and PolXBs variants the estimated Hill coefficient (*n*) was slightly higher than 1, a fact that could be indicating a weak positive cooperativity in the PolXBs-DNA interaction.

**Table 1:** Steady-state kinetic parameters of the AP-endonuclease activity of the wild-type and mutant derivatives of PolXBs.

Enzyme	*k*_*cat*_ (min^−1^)	*K*_*m*_ (nM)	Cat.eff. (min^−1^nM^−1^)	*f*
wild-type	3 ± 0.11	42 ± 4	7.2 × 10^−2^	1
F440A	4.7 × 10^−3^	30 ± 5	1.5 × 10^−4^	480
R469A	8.5 × 10^−3^ ± (3.4 × 10^−4^)	35 ± 5	2.4 × 10^−4^	300
R474A	4 × 10^−3^ ± (2.3 × 10^−4^)	77 ± 9	5.2 × 10^−4^	138
N498A	7.1 × 10^−3^ ± (3.4 × 10^−4^)	50 ± 7	1.4 × 10^−4^	514
R503A	1.1 × 10^−3^ ± (8.4 × 10^−5^)	26 ± 6	4.3 × 10^−5^	1674
K545A	1.9 × 10^−2^ ± (8.9 × 10^−3^)	70 ± 8	2.8 × 10^−4^	257
Δ325-326	0.2 ± (8.4 × 10^−3^)	116 ± 13	1.5 × 10^−3^	48

Data are means ± standard error of at least three independent experiments.

*f*: (Cat.eff.)_wt_/(Cat. Eff.)_mutant_

**Figure 4:**
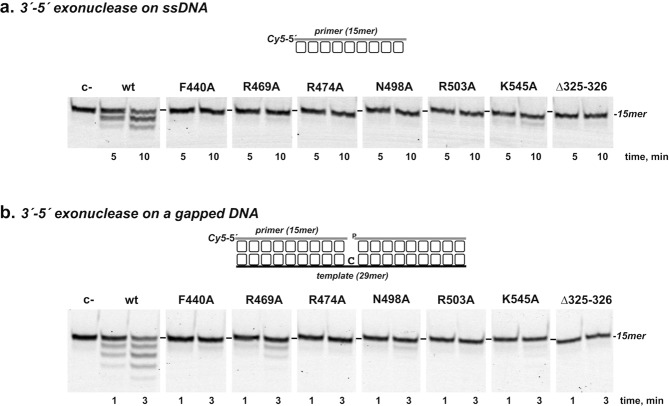
3′-5′ exonuclease activity of the PolXBs mutants. The assays were performed as described in Materials and Methods, incubating 13 nM of either the ssDNA oligonucleotide Cy5P **(a)** or the 1-nt gapped DNA substrate (**b**), with either 50 nM (**a**) or 13 nM (**b**) of either the wild-type or the specified PolXBs variant. Samples were incubated at 30 °C for the indicated times and products resolved by denaturing PAGE and visualized using a Typhoon 9410 scanner (GE Healthcare). Full length gels are presented in Fig. S5.

**Figure 5:**
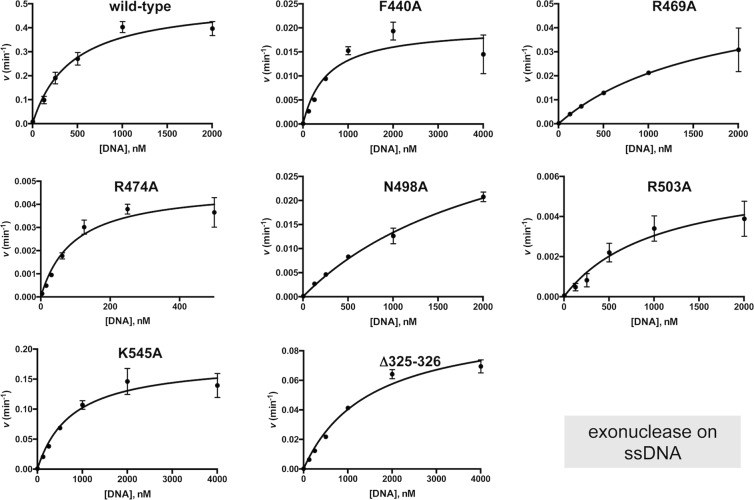
Steady-state analysis of the 3′-5′ exonuclease activity of the PolXBs derivatives. The assays were carried out as described in Materials and Methods. Graphics show the turnover values (*v* in min^−1^) plotted as a function of ssDNA concentration (^32^P-5′ labeled oligonucleotide THF-11P; see Materials and Methods). The data were fitted to the Michaelis–Menten equation by least-squares nonlinear regression and the resulting *kcat*, *Km* and catalytic efficiency (*kcat*/*Km*) values are given in Table 2.

As mentioned, the Mn^2+^-dependent 3′-5′ exonuclease activity of PolXBs enables the enzyme to process mismatched 3′-ends in gapped DNA substrates^19^. To determine whether the PHP residues studied here play any role in aiding this nucleolytic activity, mutant polymerases were subjected to 3′-5′ exonuclease assays, using 40 µM Mn^2+^ (see Supplementary Fig. S1b and Materials and Methods). As shown in Fig. 4, changes at residues Arg^474^, Phe^440^, Arg^469^, Asn^498^, Arg^503^ and Lys^545^ reduced the 3′-5′ exonuclease activity of PolXBs both, on ssDNA (Fig. 4a) and 1-nt gapped DNA substrate (Fig. 4b). Steady-state analysis of the 3′-5′ exonuclease activity of the PolXBs variants on ssDNA showed the reduction of the *k*_*cat*_ as the main cause of the drop of their catalytic efficiencies (Fig. 5 and Table 2). Mutants R469A, N498A and Δ325-326 displayed also an apparent *K*_*m*_ 4-, 6- and 4-fold higher than that of the wild-type polymerase, suggesting a defective binding of the ssDNA substrate at the PHP domain. Additionally, all the PolXBs mutants exhibited a very deficient 3′-phosphatase and phosphodiesterase activities (see Supplementary Fig. S2).

**Table 2:** Steady-state kinetic parameters of the 3′-5′ exonuclease activity of the wild-type and mutant derivatives of PolXBs.

Enzyme	*k*_*cat*_ (min^−1^)	*K*_*m*_ (nM)	Cat.eff. (min^−1^nM^−1^)	*f*
wild-type	0.51 ± (2.8 × 10^−2^)	431 ± 65	1.2 × 10^−3^	1
F440A	2 × 10^−2^ ± (1.8 × 10^−3^)	528 ± 145	3.8 × 10^−5^	32
R469A	5.8 × 10^−2^ ± (1 × 10^−2^)	1737 ± 579	3.3 × 10^−5^	36
R474A	4.8 × 10^−3^ ± (3 × 10^−4^)	94 ± 17	5.1 × 10^−5^	24
N498A	4.4 × 10^−2^ ± (4 × 10^−3^)	2318 ± 337	1.9 × 10^−5^	63
R503A	6.2 × 10^−3^ ± (9.8 × 10^−4^)	1041 ± 338	5.9 × 10^−5^	20
K545A	0.18 ± (1.1 × 10^−2^)	772 ± 132	2.3 × 10^−4^	5
Δ325-326	0.1 ± (5.5 × 10^−3^)	1500 ± 186	6.7 × 10^−5^	18

Data are means ± standard error of at least three independent experiments.

*f*: (Cat.eff.)_wt_/(Cat. Eff.)_mutant_

### Biophysical binding of PolXBs mutants to a ssDNA containing an internal abasic site

**Figure 6:**
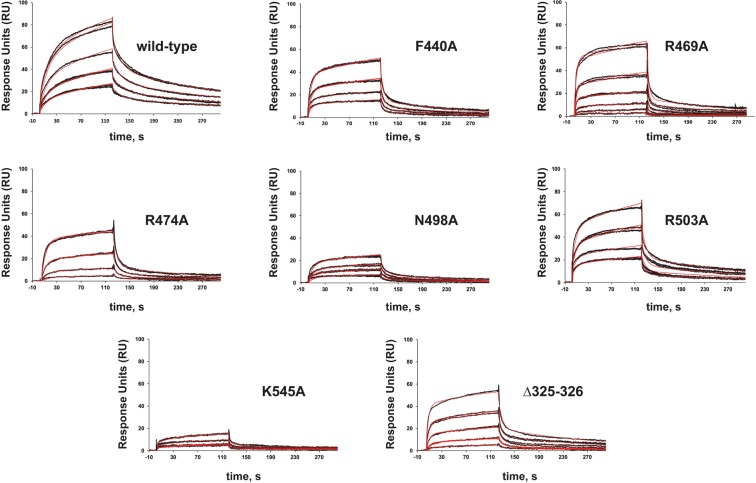
Interaction of the wild-type and mutant polymerases with an AP-containing ssDNA. Assays were carried out as described in Materials and Methods. Representative SPR sensorgrams for binding of increasing concentrations (23–300 nM) of either the wild-type or the indicated PolXBs variant to the immobilized 3′-biotinylated oligonucleotide THF-11B are shown. The black lines are sensorgrams obtained from duplicate injections of each protein concentration and the red lines depict the global fits to a 2:1 bivalent interaction model.

To ascertain whether the mutations introduced at the PolXBs PHP domain affect the binding affinity of the enzyme, we used a Surface Plasmon Resonance-based analysis. The ssDNA containing a THF moiety at the 11 position and a biotin at the 3′-end (110 RU) was immobilized onto a sensorchip SA, and varying concentrations of either wild-type or mutant polymerases were flowed over the chip to measure the binding affinity of the polymerase to DNA. Data were fitting using a bivalent analyte model as a two-step process (see Materials and Methods). As shown in Fig. 6 and Supplementary Table S2, the apparent dissociation constant (K_D1_) of F440A, R469A, R474A, N498A, R503A, K545A and Δ325-326 was reduced 3.6-, 11.4-, 1.3-, 2.9-, 2.3-, 4.3- and 1.7-fold respect to that of the wild-type polymerase, in good agreement with a role for these residues in DNA binding. Altogether, the results would indicate that these residues allow the correct orientation/stabilization of the DNA substrate when both, the 3′ terminus and the AP site have to be placed at the catalytic site of the PHP domain.

### Effect of site-directed mutations at the PHP domain in the polymerization activity of PolXBs

**Figure 7:**
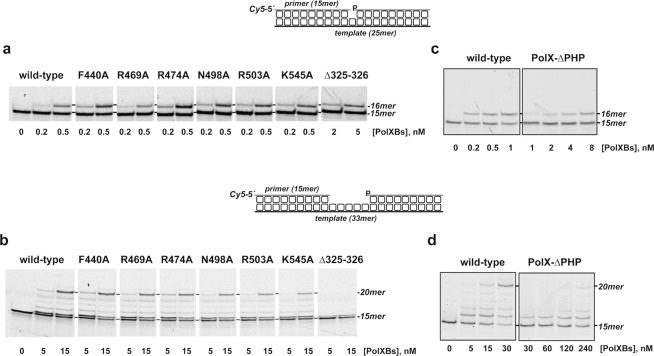
DNA polymerization activity. (**a,b**) DNA polymerization activity on gapped DNA of PolXBs variants. Assays were performed as described in Materials and Methods, incubating 10 nM of either a 1-nt (**a**) or a 5-nt (**b**) gapped DNA with the indicated polymerase concentration. Samples were incubated at 30 °C for either 1 min (**a**) or 10 min (**b**). Products were resolved by denaturing PAGE and visualized with a Typhoon 9410 scanner (GE Healthcare) **(c,d)** DNA polymerization activity of the Polβ-like core of PolXBs on gapped DNA. Assays were performed as described in Materials and Methods, incubating either 4 nM of a 1-nt gapped DNA (**c**) or 10 nM of a 5-nt gapped DNA (**d**) with the indicated amount of either the wild-type or the ΔPHP PolXBs variant^19^. Samples were incubated at 30 °C for 10 min and analyzed as described above. Full length gels are presented in Fig. S6.

PolXBs is involved in repairing DNA lesions due to its inherent capacity to accommodate itself in the short gaps that arise in the course of DNA repair processes. To ascertain whether the residues studied here play any role in the polymerization activity of PolXBs, we analyzed the gap-filling ability of the mutant proteins using either a 1-nt (Fig. 7a) and a 5-nt (Fig. 7b) gapped DNA substrate (see Materials and Methods). As it can be observed, the point mutant derivatives displayed a nearly wild-type phenotype on both substrates, indicating that during polymerization those residues are not involved in making contacts with the DNA substrate, allowing also to rule out a general misfolding of the mutant polymerases as responsible for their low nucleolytic activities described above.

Interestingly, the polymerization activity of mutant Δ325-326 was 10-fold lower than that of the wild-type protein in the 1-nt gapped DNA, being unable to give rise to products longer than +1 in the longest gapped substrate. This result would suggest that polymerization relies on an adequate orientation of the PHP domain respect to the polymerization one, either to confer a proper conformation of the latter to accomplish nucleotide insertion or to allow PHP domain to establish additional DNA contacts to stabilize the DNA polymerase/DNA complex. Therefore, we evaluated the effect of the PHP domain in the nucleotide insertion activity of the polymerase. To that, the deletion mutant PolXBs-ΔPHP, lacking the C-terminal PHP domain (residues 315-570^19^), was analyzed in its competence to perform the gap-filling reaction. As shown in Fig. 7, the absence of the C-terminal PHP domain greatly impaired the gap-filling ability of PolXBs on both, the 1-nt (Fig. 7c) and the 5-nt (Fig. 7d) gapped substrates.

### Intermolecular switching between the PHP and polymerization active sites

**Figure 8:**
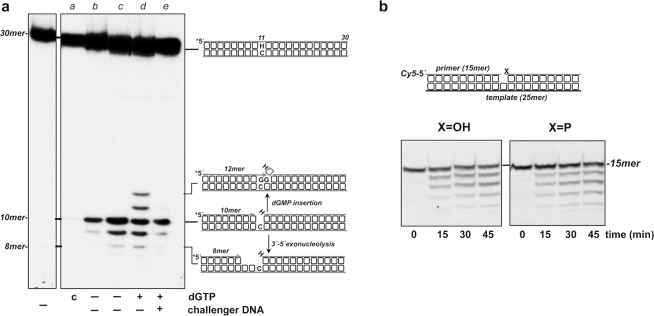
(**a**) Transference of the 3′-terminus from the PHP to the polymerization active site. Assays were performed as described in Materials and Methods, by incubating the hybrid THF-11/THF-11C (see Materials and Methods) with 25 nM of the wild-type PolXBs and 3.2 mM MnCl_2_ for 30 min (lane *b*) followed by an additional 30 min incubation in the absence (lane *c*) or presence (lanes *d* and *e*) of challenger DNA, and either in the absence (lanes *c* and *d*) or presence (lane *e*) of 500 µM dGTP. Lane *a*, control of the effectiveness of the trapping DNA. **(b)** 3′-5′ exonuclease activity of PolXBs on phosphorylated and unphosphorylated gapped substrates. Assays were performed as described in Materials and Methods, incubating 10 nM PolXBs with 10 nM of either the phosphorylated or unphosphorylated 1-nt gapped DNA. Products were resolved by denaturing PAGE and visualized with a Typhoon 9410 scanner (GE Healthcare). Full gels are presented in Fig. S7.

Previous studies showed the functional coordination of the AP-endonuclease and polymerization activities of bacterial PolXs, and consequently of the PHP and polymerization domains, which enables the polymerase to recognize, incise, and further restore *in vitro* the genetic information of the damaged DNA back to its original state in the absence of additional factors^20^. Thus, once the AP-endonuclease hydrolyzes the phosphodiester bond at the 5′ side of the AP site, the resulting 3′-OH end has to be relocated at the polymerization active site to let the subsequent gap-filling reaction. To determine the type of primer terminus transference between the two catalytic sites, we analyzed the nucleotide insertion following the processing of an internal AP site under conditions in which a single association event is allowed to occur (see Fig. 8a). To that, we incubated the PolXBs with the 5′-labeled dsDNA harboring a THF at the 11 position in the presence of Mn^2+^ ions to allow the AP-endonucleolysis to proceed (lane *b*). To promote further elongation of the newly generated 3′-temini, samples were incubated for additional 30 min either in the absence (lane *c*) or presence (lanes *d* and *e*) of dGTP, the nucleotide complementary to the templating one opposite the AP site (see scheme in Fig. 8a), and in the absence (lanes *c* and *d*) or presence (lane *e*) of a molar excess of activated calf-thymus DNA. The activated DNA acts as competitor to trap the DNA polymerase molecules non-associated with DNA before starting the reaction, and to prevent the reassociation with the DNA substrate of those DNA polymerase molecules that dissociate at later stages of the reaction process (see Materials and Methods). As shown, in the absence of both, challenging DNA and dGTP, the AP-endonuclease activity of PolXBs gave rise mainly to a 10mer product (lane *c*). The 9mer product results from the 3′-5′ exonucleolytic degradation of the incised AP site by PolXBs. Whereas in the absence of the trapping DNA PolXBs inserted dGMP onto the resulting 3′-OH end (lane *d*), its presence precluded the primer extension (lane *e*), the AP-endonuclease and the 3′-5′ exonuclease being the only activities detected. As a control of the effectiveness of the DNA trap, when the challenger DNA was simultaneously added with the labeled substrate as part of the enzyme-DNA preincubation mixture, no activity was detected (lane *a*). This result allows us to conclude that the switching of the 3′-OH end between the PHP and the polymerization active sites is accomplished intermolecularly. Thus, once PolXBs either hydrolyzes the AP site or releases a 3′-dNMP, dissociates from the DNA to allow the allocation of the resulting 3′-OH end at the polymerization active site of another PolXBs molecule to accomplish the gap-filling step.

The above results could be suggesting an alternative binding mode of PolXBs when its nucleolytic activities have to process the DNA. Despite most PolXs are distributive enzymes when acting on template-primer molecules, they accomplish processive filling of short DNA gaps. Such a processive gap filling is structurally and functionally related to the presence of the N-terminal 8-kDa domain and strongly favored by the presence of a 5′-phosphate (P) group at the end of the gap. PolXBs, as most PolXs interacts with the 5′-P group which confers a higher DNA binding stability, increasing the catalytic efficiency of the gap filling reaction^28^. To test the effect of the 5′-P group in the 3′-5′ exonuclease activity we carried out time course experiments using as substrate a DNA molecule harboring a 1-nt gap flanked by a 3′-OH group and either a 5′-OH or a 5′-P moiety. As it can be observed in Fig. 8b, the 3′-5′ exonuclease activity of PolXBs does not discriminate between phosphorylated and unphosphorylated gaps. This result suggests that during exonucleolysis the Polβ-like core is not accommodated in the gap as it is during the polymerization reaction.

## Discussion

PolXBs displays the general enzymatic characteristics shown by most PolXs. Thus, the enzyme inserts nucleotides in a template directed manner, shows a distributive polymerization pattern, and uses preferentially 5′-phosphorylated gapped DNA substrates^28^. Whereas the above-mentioned characteristics rely on the N-terminal Polβ-like core, PolXBs shares with most of the bacterial/archaeal PolXs a C-terminal PHP domain that has an intrinsic Mn^2+^-dependent 3′-5′ exonuclease activity that allows the polymerase to resect unannealed 3′-ends; an intrinsic AP-endonuclease activity that capacitates PolXBs to recognize and incise at an AP site to further restore the original nucleotide^20^; and a Mn^2+^-dependent 3′-phosphodiesterase and 3′-phosphatase activities that together with the 3′-5′ exonuclease let the polymerase to perform gap-filling once the damaged 3′-termini are processed^23^. Thus, PolXBs could act as a Swiss army knife dealing with AP sites or 3′-damaged ends to restore the original (non-damaged) nucleotide. The higher sensitivity to oxidative agents displayed by *B. subtilis* strains after disrupting the *yshC* gene that codes for PolXBs has allowed to demonstrate the involvement of the polymerase in DNA repair pathways during the life cycle of the bacterium^26^.

Previous sequence alignments of the PHP domain of bacterial/archaeal PolXs, together with site directed mutagenesis and structural studies allowed the identification of the nine highly conserved residues that make up the catalytic site^17,19–24,29^. Those residues coordinate three metal ions^24,29^ and catalyze the nucleolytic activities of these polymerases: AP-endonuclease, 3′-5′ exonuclease, 3′-phophodiesterase and 3′-phosphatase^19–23^. Besides the catalytic residues, we have shown here the presence of additional and highly conserved residues along the PHP domain of bacterial/archaeal PolXs, and corresponding to PolXBs residues Arg^474^, Phe^440^, Arg^469^, Asn^498^, Arg^503^ and Lys^545^ that in the tertiary structure form a path towards the catalytic site, suggesting a role in contacting the DNA during its nucleolytic processing. PolXBs variants at those residues exhibited very reduced AP-endonuclease and 3′-5′ exonuclease activities due to both, a reduced *kcat*, and a diminished DNA binding capacity, unveiling the importance of those residues in the proper binding of the DNA at the PHP catalytic site to allow further processing of the substrate. Besides the bacterial/archaeal PolXs, the bacterial replicative DNA polymerases also contain a PHP domain^17^ that can be predicted to be either in an active or inactive conformation depending on either the presence or not of the nine catalytic residues^30^. Thus, in those bacterial replicases whose proofreading function resides in the DnaQ-like exonuclease, as in *E. coli* Pol III, the PHP domain has been suggested to play a structural role, modulating the stability and activity of the polymerase^31^, as well as it has been demonstrated to control the DNA extension rate by pyrophosphate hydrolysis^32,33^. By contrast, in those replicative DNA polymerases lacking the prototypical DnaQ-like exonuclease, as in the replicases from *Mycobacterium tuberculosis*^30,34^ and *T. thermophilus*^35^, the PHP domain contains the 3′-5′ exonuclease activity that proofreads the misinserted nucleotides. The recent determination of the crystallographic structure of the DNA polymerase DnaE1 from *M. tuberculosis* revealed the presence of a narrow groove at the N-terminal PHP domain that would channel a mispaired 3′-terminus towards the buried catalytic site^34^. In contrast, and as mentioned before, the catalytic residues of PolX PHP domains are solvent exposed and placed close to the molecular surface of the domain without any evident cleft that could assist the binding of the ssDNA substrate. The dramatic drop in the nucleolytic activities observed with each of the individual PolXBs variants studied here connotes that the superficial binding at the PHP domain requires the orchestrated and simultaneous interaction of the DNA with all the residues that form the electropositive path. In addition, the PolXBs variants displayed a nearly wild-type efficiency in filling short gaps, indicating that the above-mentioned DNA binding residues do not establish interactions with the DNA when the polymerase is in a polymerization mode. Altogether, the results suggest a common DNA binding site at the PHP domain for the nucleolytic activities of the enzyme. Interestingly, the shortening of the linker that connects the C-terminal PHP domain and the N-terminal Polβ-like core in the Δ325-326 mutant also diminished the nucleolytic activities of the enzyme, although to a lower extent than the single mutant derivatives. This result indicates that both domains are required to be properly oriented one to each other to perform the nucleolytic activities in a proficient manner, in agreement with previous results that showed a more than a 10-fold drop in the AP-endonuclease activity of the independently expressed PHP domain^20^.

As shown in Fig. 1b, the use of the crystallographic structure of ttPolX ternary complex with a 1nt-gap^24^ allowed to model PolXBs^36^. The folding of the Polβ-like core would allow the catalysis of the polymerization reaction where the palm, thumb and fingers wrap the upstream portion of the gapped molecule, the 8-kDa domain interacts with the downstream 5′-P group, and the PHP domain forms a right angle with the DNA (see Fig. 1b). Here, we have shown the importance of the PHP domain in the polymerization reaction, as both, the deletion of this domain and the shortening of the linker region in mutant Δ325-326 impaired polymerization. Although in the ternary complexes of ttPolX the PHP domain does not make contacts with the DNA, it establishes stacking interactions with residues of the palm, 8-kDa and thumb subdomains^24^, suggesting the importance of the PHP domain in maintaining the polymerization competent structure of the Polβ-like *core*. If this were also the catalytically competent conformation for the nucleolytic activities, and considering the arrangement of the PHP DNA binding residues (Fig. 1c), the primer terminus should be melted 3-4 nt to bind the PHP and to reach the catalytic site. However, it would be rather difficult to envision how an internal AP site embedded in a dsDNA could reach the PHP active site. In addition, the binding of a dsDNA containing an internal AP site to the Polβ-like *core* would demand large structural rearrangements because of the absence of internal nicks or short gaps. That the presence of a 5′-phosphorylated gap did not stimulate the 3′-5′ exonuclease activity suggests that at least during exonucleolysis the 8-kDa domain does not bind the 5′-phosphate moiety. Consequently, the relative orientation of the N-terminal Polβ-like *core* and the C-terminal PHP domain observed in the ternary complex with a gapped DNA substrate could not allow the proper coordination among the synthetic and nucleolytic activities of the polymerase. The presence of an only dsDNA binding cleft raises the question of how the DNA accesses to the PHP active site. The fact that substitutions at DNA binding residues of the Polβ-like *core* of PolXBs and PolXDr not only affected the polymerization capacity of the enzyme, but also hampered the PHP-dependent nucleolytic activities^36,37^, together with the interaction observed to occur between the polymerization and PHP domains of ttPolX when they were independently expressed^21^, led us to propose that the PHP domain would rotate towards the Polβ-like *core* to reach both, an AP site embedded in a dsDNA and a mispaired 3′-end, a major structural change that would be aided by the long linker located between both domains^36^. However, this model would imply that the common electropositive path of the PHP domain would bind in an opposed polarity a 3′-terminus during exonucleolysis and the AP-containing strand during AP endonucleolysis.

The crystallographic resolution of the apo PolXDr showed that the Pol-β like *core* did not adopt the semi open closed right hand structure observed in most DNA polymerases but, instead, was arranged in a completely extended conformation^29^. In such structures, the fingers and 8-kDa N-domains were swung out by 90° compared with the Polβ conformation, implying that the DNA binding region of the 8-kDa domain and of the palm subdomain are on opposite sides of the protein surface^29^. Importantly, the C-terminal PHP domain stabilized the stretched conformation of the polymerase. Thus, to accomplish the polymerization reaction the enzyme must undergo drastic structural rearrangements to adopt the canonical Polβ-like arrangement. The structural superposition of the binary (with an incoming nucleotide) and ternary complexes (with either a primer/template structure or a 1nt-gapped DNA) of ttPolX showed that the palm, thumb, and PHP domains superimpose perfectly^24^. However, only the presence of a downstream strand in the gapped molecule provoked a large shifting of the 8-kDa and fingers subdomains, the Polβ-like *core* adopting the classical right hand-like conformation to allow the insertion of the incoming nucleotide whereas the polymerase/DNA complex is stabilized through the interaction between the downstream 5′-P and the 8-kDa domain. Those structures, together with the results presented here suggest that the more stretched conformation of these polymerases in the absence of a gapped DNA molecule and an incoming nucleotide could represent an alternative catalytically competent state of the enzyme during AP endonucleolysis and 3′-5′ exonucleolytic resection, where the DNA substrates could be stabilized by the PHP residues described here to gain direct access to the PHP catalytic site, as well as by the specific HhH motif present in bacterial/archaeal PolXs^36^. Thus, the dramatic conformational changes of the N-terminal 8-kDa and fingers subdomains in response to the type of DNA substrate would confer bacterial PolXs with the high degree of functional and structural flexibility required to coordinate the synthetic and degradative activities of these enzymes. Finally, we have shown that the 3′-OH end that results after the endonucleolytic cleavage of an AP site is intermolecularly switched from the PHP active site to the polymerization one. Such enzyme/DNA dissociation could be necessary to allow the protein to go from a nucleolyticaly competent conformation to a polymerization competent Polβ-like arrangement.

## Methods

### Proteins, reagents and oligonucleotides

Unlabeled nucleotides were purchased from GE Healthcare. [γ^32^P]ATP was obtained from Perkin Elmer Life Sciences. Activated calf thymus DNA was from Sigma-Aldrich. Wild-type PolXBs and PolXBs-ΔPHP were expressed and purified as described^19,28^. Oligonucleotides were purchased from Integrated DNA Technologies (sequences are listed in Table 3). When indicated, oligonucleotides were radiolabeled at the 5′ end using [γ^32^P]ATP (3000 Ci/mmol) and T4 polynucleotide kinase (New England Biolabs). Substrates were annealed as described^36^, in the presence of 60 mM Tris-HCl (pH 7.5) and 0.2 M NaCl at 80 °C for 5 min before slowly cooling to room temperature.

**Table 3:** Oligonucleotides used in this study.

Name	Sequence (5′-3′)
P	GATCACAGTGAGTAC
3′-P	GATCACAGTGAGTAC(P)
3′-PG	GATCACAGTGAGTAC(PG)
Cy5P	(Cy5)GATCACAGTGAGTAC
DOH	AACGACGGCCAGT
DowP	(P)AACGACGGCCAGT
T33	ACTGGCCGTCGTTCTATTGTACTCACTGTGATC
T29	ACTGGCCGTCGTTCGTACTCACTGTGATC
THF-11	TGACTGCATA**H**GCATGTAGACGATGTGCAT
THF-11B	TGACTGCATA**H**GCATGTAGACGATGTGCAT-Bio
THF-11*	TGACTGCATA**H**GCATGTAGACGATGTGC*A*T
THF-11C	ATGCACATCGTCTACATGCCTATGCAGTCA
THF-19	(Cy5)GTACCCGGGGATCCGTAC**H**GCGCATCAGCTGCAG

Cy5: 1,1′-bis(3-hydroxypropyl)-3,3,3′,3′-tetramethylindodicarbocyanine dye.

H: tetrahydrofuran.

Bio: 3′-biotin.

*Phosphorothioate bond.

(P): phosphate group.

(PG): phosphoglycolate group.

### Site-directed mutagenesis of PolXBs

PolXBs variants F440A, R469A, R474A, N498A, R503A, K545A and Δ325-326 were made with the QuickChange site-directed mutagenesis kit (Stratagene), using as template for the mutagenesis reaction the plasmid pET28-PolXBs that contains the PolXBs gene^28^. Expression and purification of the mutant proteins were performed essentially as described for the wild-type PolXBs^28^ (see Supplementary Fig. S3).

### DNA polymerization assays

The assays were performed essentially as described^28^. Thus, the reaction mixture (12.5 µl) containing 50 mM Tris-HCl (pH 7.5), 8 mM MgCl_2_, 1 mM DTT, 4% glycerol, 0.1 mg/ml Bovine Serum Albumin (BSA), 100 µM of dNTPs, 10 nM of either the 5-nt gapped DNA (obtained by hybridization of oligonucleotides Cy5P, T33 and DowP; see Table 3) or the 1-nt gapped molecule (obtained by hybridization of oligonucleotides Cy5P, T29 and DowP; see Table 3), and enzyme as indicated were incubated at 30 °C for either 10 min (5 nt gap) or 1 min (1 nt gap). The reactions were stopped by adding EDTA to 10 mM. The reaction products were resolved by 7 M urea-20% PAGE and visualized with a Typhoon 9410 scanner (GE Healthcare).

### AP-endonuclease assays

The assays were performed essentially as described^36^. Thus, the reaction mixtures (12.5 µl) containing 50 mM Tris-HCl (pH 7.5), 40 µM MnCl_2_, 1 mM DTT, 4% glycerol, 0.1 mg**/**mL BSA, 4 nM of either the ^32^P-5′ labeled oligonucleotide THF-11 that harbors an AP site at the 11 position (see Table 3) or the hybrid ^32^P-5′-THF-11/THF-11C (see Table 3) and enzyme as specified were incubated at 30 °C for either 2 min (ssDNA) or 5 min (dsDNA). The reactions were stopped by adding EDTA to 10 mM. The products were resolved by 7 M urea-20% PAGE and visualized by autoradiography. *Steady-state AP endonuclease assays:* The incubation mixtures contained, in 12.5 μl, 50 mM Tris-HCl (pH 7.5), 40 µM MnCl_2_, 1 mM DTT, 4% glycerol, 0.1 mg**/**mL BSA and ^32^P-5′ labeled oligonucleotide THF-11P (5–325 nM). This oligonucleotide harbors two phosphorothioate bonds to avoid 3′-5′ exonucleolytic degradation. The reaction was started by adding either 2 nM of the wild-type PolXBs or 100 nM of PolXBs variants. After incubation for 5 min (wild-type and Δ325-326 mutant), 15 min (mutant R469A) and 30 min (mutants F440A, R474A, N498A, R503A and K545A), reactions were quenched by adding EDTA to 10 mM. The products were resolved by 7 M urea-20% PAGE and visualized by autoradiography. Gel band intensities were quantified using ImageQuant TL software (GE Healthcare). Only those reactions that fell within the linear range of substrate utilization (<20% substrate) were used for analysis. The turnover values (*v* in min^−1^) were calculated as described in^38^, and plotted as a function of DNA concentration. Steady-state kinetic parameters, *V*_max_ and *K*_M_, were determined by least-squares nonlinear regression fitting of the data to the Michaelis–Menten equation.

### 3′-5′ exonuclease assays

The reaction mixtures (12.5 µl) containing 50 mM Tris-HCl (pH 7.5), 40 µM MnCl_2_, 1 mM DTT, 4% glycerol, 0.1 mg/ml BSA, 13 nM of either the oligonucleotide Cy5P or the 1-nt gapped molecule (obtained by hybridization of oligonucleotides Cy5P, T29 and DowP; see Table 3) and either 52 nM (ssDNA) or 13 nM (gapped molecule) of the specified polymerase were incubated at 30 °C for the indicated times. The reactions were stopped by adding EDTA to 10 mM. Reaction products were resolved by 7 M urea-20% PAGE and visualized with a Typhoon 9410 scanner (GE Healthcare). *Steady-state 3*′*-5*′ *exonuclease assays:* The reaction mixture (12.5 µl) contained 50 mM Tris-HCl (pH 7.5), 40 µM MnCl_2_, 1 mM DTT, 4% glycerol, 0.1 mg**/**mL BSA and ^32^P-5′ labeled oligonucleotide P (4–4004 nM). The reaction was started by adding either 50 nM of the wild-type PolXBs or 100 nM of PolXBs variants. After incubation for 3 min (wild-type) and 60 min (mutant derivatives), reactions were quenched by adding EDTA to 10 mM. Reaction products were resolved by 7 M urea-20% PAGE and visualized by autoradiography. The intensities of the gel bands were quantified with the ImageQuant TL software (GE Healthcare) and analyzed as described for the AP-endonuclease assays. Only those reactions that fell within the linear range of substrate utilization (<20% substrate) were used for analysis.

### 3′-phosphatase/phosphodiesterase activity

The assays were performed essentially as described^23^. Thus, the reaction mixture (12.5 µl) contained 50 mM Tris-HCl (pH 7.5), 40 µM MnCl_2_, 1 mM DTT, 4% glycerol, 0.1 mg**/**ml BSA, 1 nM of either the ^32^P-5′ labeled oligonucleotide 3′-P (for the analysis of the 3′-phosphatase activity; see Table 3) or the ^32^P-5′ labeled oligonucleotide 3′-PG (for the analysis of the 3′-phosphodiesterase activity) and either 2.5 nM or 5 nM of PolXBs, respectively. Samples were incubated at 30 °C for the indicated times and the reactions quenched by adding EDTA to 10 mM. Reactions products were resolved by 7 M urea-20% PAGE and visualized by autoradiography.

### Binding assays of DNA polymerases to an AP-containing oligonucleotide

SPR experiments were performed in a biosensor Biacore 3000 (GE Healthcare). The 29-nt oligonucleotide THF-11B (see Table 3) with a 3′ terminal biotin group was immobilized on a streptavidin-coated sensor chip (SA) at a flow rate of 10 µl/min (110 RU captured). Reference surface was a flow cell left blank as control. Tris-HCl 50 mM, 0.005% Surfactant P20, pH 7.5 was used as running buffer. The binding analysis with the wild type DNA polymerase and their different mutants was carried out at 25 °C with a flow rate of 30 µl/min, where each injection of analyte was performed in duplicated within each assay. Binding was also tested at 100 µl/min and the slope of the binding curves did not show mass transport limitations. The sensor surface was regenerated using a 30 s pulse of 800 mM sodium chloride. Data were collected for 120 s of the association phase and 180 s of the dissociation phase. Sensorgrams with different concentrations of analyte (23–80 nM for the wild-type and mutants F440A, N498A, R503A and K545A; 25–200 nM for mutant R474A; and 25–300 nM for mutants R469A and Δ325-326) were overlaid, aligned and analyzed with BIAevaluation Software 4.1. All data set were processed using a double-referencing method^39^ and the binding curves were fit using a bivalent analyte model.

### Transference of the primer terminus from the PHP to the polymerization active site

The reaction mixture (12.5 µl) contained 50 mM Tris-HCl (pH 7.5), 1 mM dithiothreitol, 4% glycerol, 0.1 mg/ml BSA, 4 nM of the ^32^P 5′-labeled hybrid THF-11/THF-11C and 25 nM of wild-type PolXBs. After incubation for 30 min at 30 °C, in the presence of 3.2 mM MnCl_2_ to allow AP-endonucleolysis, polymerization reaction was started by the simultaneous addition of 500 µM dGTP and 3 µg of activated calf thymus DNA as trap. The samples were incubated for additional 30 min at 30 °C. As a control of the effectiveness of the competitor DNA, PolXBs was incubated simultaneously with the labeled DNA used as substrate and 3 µg of the activated DNA. The reactions were stopped by adding EDTA to 10 mM. The products were resolved by 7 M urea-20% PAGE and visualized by autoradiography.

## Supplementary information

Supplementary Information

## References

[1] Hoeijmakers JH (2001). Genome maintenance mechanisms for preventing cancer. Nature.

[2] Barnes DE, Lindahl T (2004). Repair and genetic consequences of endogenous DNA base damage in mammalian cells. Annu. Rev. Genet..

[3] Almeida KH, Sobol RW (2007). A unified view of base excision repair: lesion-dependent protein complexes regulated by post-translational modification. DNA repair.

[4] Braithwaite EK (2005). DNA polymerase lambda mediates a back-up base excision repair activity in extracts of mouse embryonic fibroblasts. J. Biol. Chem..

[5] Fan W, Wu X (2004). DNA polymerase lambda can elongate on DNA substrates mimicking non-homologous end joining and interact with XRCC4-ligase IV complex. Biochem. Biophys. Res. Commun..

[6] García-Díaz M, Bebenek K, Kunkel TA, Blanco L (2001). Identification of an intrinsic 5′-deoxyribose-5-phosphate lyase activity in human DNA polymerase lambda: a possible role in base excision repair. J Biol Chem.

[7] Lecointe F, Shevelev IV, Bailone A, Sommer S, Hübscher U (2004). Involvement of an X family DNA polymerase in double-stranded break repair in the radioresistant organism *Deinococcus radiodurans*. Mol. Microbiol..

[8] Lee JW (2004). Implication of DNA polymerase lambda in alignment-based gap filling for nonhomologous DNA end joining in human nuclear extracts. J. Biol. Chem..

[9] Mahajan KN, Nick McElhinny SA, Mitchell BS, Ramsden DA (2002). Association of DNA polymerase mu (pol mu) with Ku and ligase IV: role for pol mu in end-joining double-strand break repair. Mol. Cell Biol..

[10] Matsumoto Y, Kim K (1995). Excision of deoxyribose phosphate residues by DNA polymerase beta during DNA repair. Science.

[11] Moon AF (2007). The X family portrait: Structural insights into biological functions of X family polymerases. DNA Repair (Amst).

[12] Nick McElhinny SA (2005). A gradient of template dependence defines distinct biological roles for family X polymerases in nonhomologous end joining. Mol. Cell.

[13] Yamtich J, Sweasy JB (2010). DNA polymerase family X: function, structure, and cellular roles. Biochim. Biophys. Acta.

[14] Hübscher U, Maga G, Spadari S (2002). Eukaryotic DNA polymerases. Annu. Rev. Biochem..

[15] Beard WA, Wilson SH (2000). Structural design of a eukaryotic DNA repair polymerase: DNA polymerase beta. Mutat. Res..

[16] Bork P (1997). A superfamily of conserved domains in DNA damage-responsive cell cycle checkpoint proteins. FASEB J..

[17] Aravind L, Koonin EV (1998). Phosphoesterase domains associated with DNA polymerases of diverse origins. Nucleic Acids Res..

[18] Ramadan K, Shevelev I, Hubscher U (2004). The DNA-polymerase-X family: controllers of DNA quality?. Nat. Rev. Mol. Cell Biol..

[19] Baños B, Lázaro JM, Villar L, Salas M, de Vega M (2008). Editing of misaligned 3′-termini by an intrinsic 3′-5′ exonuclease activity residing in the PHP domain of a family X DNA polymerase. Nucleic Acids Res..

[20] Baños B, Villar L, Salas M, de Vega M (2010). Intrinsic apurinic/apyrimidinic (AP) endonuclease activity enables *Bacillus subtilis* DNA polymerase X to recognize, incise, and further repair abasic sites. Proc. Natl. Acad. Sci. USA.

[21] Nakane S, Nakagawa N, Kuramitsu S, Masui R (2009). Characterization of DNA polymerase X from *Thermus thermophilus* HB8 reveals the POLXc and PHP domains are both required for 3′-5′ exonuclease activity. Nucleic Acids Res..

[22] Nakane S, Nakagawa N, Kuramitsu S, Masui R (2012). The role of the PHP domain associated with DNA polymerase X from *Thermus thermophilus* HB8 in base excision repair. DNA Repair (Amst).

[23] Zafra O, Pérez de Ayala L, de Vega M (2017). The anti/syn conformation of 8-oxo-7,8-dihydro-2′-deoxyguanosine is modulated by *Bacillus subtilis* PolX active site residues His255 and Asn263. Efficient processing of damaged 3′-ends. DNA Repair (Amst).

[24] Nakane, S., Ishikawa, H., Nakagawa, N., Kuramitsu, S. & Masui, R. The Structural Basis of the Kinetic Mechanism of a Gap-Filling X-Family DNA Polymerase That Binds Mg(2+)-dNTP Before Binding to DNA. *J. Mol. Biol*. **417** (2012). 10.1016/j.jmb.2012.01.025 22306405

[25] Teplyakov A (2003). Crystal structure of the *Escherichia coli* YcdX protein reveals a trinuclear zinc active site. Proteins.

[26] Barajas-Ornelas RC (2014). Error-prone processing of apurinic/apyrimidinic (AP) sites by PolX underlies a novel mechanism that promotes adaptive mutagenesis in *Bacillus subtilis*. J. Bacteriol..

[27] Johnson ML (2000). Mathematical modeling of cooperative interactions in hemoglobin. Methods Enzymol..

[28] Baños B, Lázaro JM, Villar L, Salas M, de Vega M (2008). Characterization of a *Bacillus subtilis* 64-kDa DNA polymerase X potentially involved in DNA repair. J. Mol. Biol..

[29] Leulliot N (2009). The family X DNA polymerase from *Deinococcus radiodurans* adopts a non-standard extended conformation. J. Biol. Chem..

[30] Rock JM (2015). DNA replication fidelity in *Mycobacterium tuberculosis*is mediated by an ancestral prokaryotic proofreader. Nat. Genet..

[31] Barros T (2013). A structural role for the PHP domain in *E. coli*DNA polymerase III. BMC Struct. Biol..

[32] Lapenta F (2016). *Escherichia coli*DnaE Polymerase Couples Pyrophosphatase Activity to DNA Replication. PLoS One.

[33] McHenry CS (2011). DNA replicases from a bacterial perspective. Annu. Rev. Biochem..

[34] Baños-Mateos S (2017). High-fidelity DNA replication in *Mycobacterium tuberculosis*relies on a trinuclear zinc center. Nat Commun.

[35] Stano NM, Chen J, McHenry CS (2006). A coproofreading Zn(2+)-dependent exonuclease within a bacterial replicase. Nat. Struct. Mol. Biol..

[36] Baños B, Villar L, Salas M, de Vega M (2012). DNA stabilization at the *Bacillus subtilis*PolX core-a binding model to coordinate polymerase, APendonuclease and 3′-5′ exonuclease activities. Nucleic Acids Res..

[37] Blasius M, Shevelev I, Jolivet E, Sommer S, Hübscher U (2006). DNA polymerase X from *Deinococcus radiodurans*possesses a structure-modulated 3′–>5′ exonuclease activity involved in radioresistance. Mol. Microbiol..

[38] O’Flaherty, D. K. & Guengerich, F. P. Steady-state kinetic analysis of DNA polymerase single-nucleotide incorporation products. Curr. Protoc. Nucleic Acid Chem. **59**, 7 21 21–13, 10.1002/0471142700.nc0721s59 (2014). 10.1002/0471142700.nc0721s59 PMC4274652 25501593

[39] Myszka DG (2000). Kinetic, equilibrium, and thermodynamic analysis of macromolecular interactions with BIACORE. Methods Enzymol..

[40] Arnold K, Bordoli L, Kopp J, Schwede T (2006). The SWISS-MODEL workspace: a web-based environment for protein structure homology modelling. Bioinformatics.

[41] Biasini M (2014). SWISS-MODEL: modelling protein tertiary and quaternary structure using evolutionary information. Nucleic Acids Res..

[42] Guex N, Peitsch MC, Schwede T (2009). Automated comparative protein structure modeling with SWISS-MODEL and Swiss-PdbViewer: a historical perspective. Electrophoresis.

[43] Kiefer F, Arnold K, Kunzli M, Bordoli L, Schwede T (2009). The SWISS-MODEL Repository and associated resources. Nucleic Acids Res..

