# Supplementary material for: An array of basic residues is essential for the nucleolytic activity of the PHP domain of bacterial/archaeal PolX DNA polymerases
Source: Sci Rep. 2019 Jul 9;9:9947. doi: 10.1038/s41598-019-46349-8 (PMC6616362; doi:10.1038/s41598-019-46349-8)
Supplement: Supplementary file 1 — Supplementary Information [file 41598_2019_46349_MOESM1_ESM.pdf]

Supplementary Information

An array of basic residues is essential for the nucleolytic activity of the PHP domain of bacterial/archaeal PolX DNA polymerases

Guillermo Rodríguez[1], **María Teresa Martín**[2] **and Miguel de Vega**[1,*]

[1]Centro de Biología Molecular "Severo Ochoa" (Consejo Superior de Investigaciones Científicas-Universidad Autónoma de Madrid), Nicolás Cabrera 1, 28049 Madrid, Spain.

[2]Centro Nacional de Biotecnología (Consejo Superior de Investigaciones Científicas), Darwin 3, 28049 Madrid, Spain

*To whom correspondence should be addressed. Tel: +34 911964717; Fax: +34 911964420; Email: mdevega@cbm.csic.es.

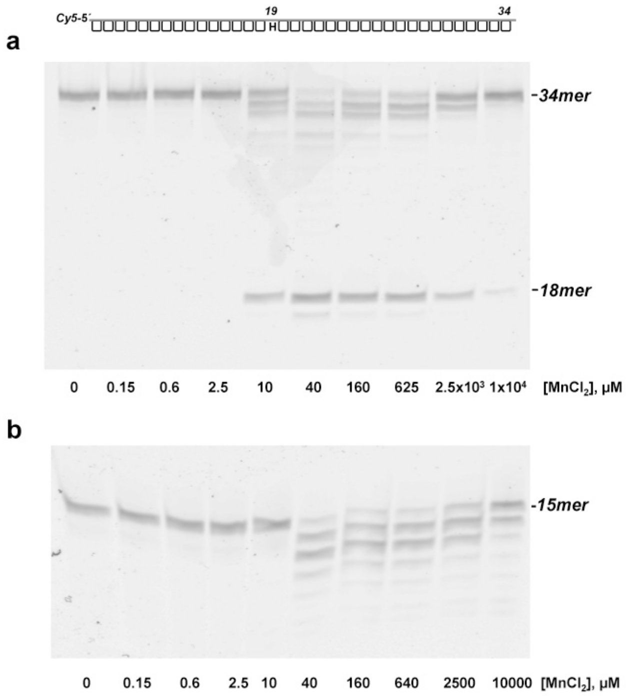

Supplementary Figure S1. (a) *Determination of the optimum Mn^{2+} concentration for the AP-endonuclease activity of PolXBs*. The assay was performed as described in Materials and Methods, in the presence of 13 nM of the AP-containing oligonucleotide THF-19 (see Table 1), 6.25 nM PolXBs and the indicated concentrations of Mn^{2+} ions. After incubation for 2 min at 30 ºC, reactions were stopped by adding 10 mM EDTA. Samples were analyzed by 7 M urea-20% PAGE and visualized using a Typhoon 9410 scanner (GE Healthcare). **(b)** *Determination of the optimum Mn^{2+} concentration for the 3´-5´ exonuclease activity of PolXBs*. The assay was performed as described in Materials and Methods, in the presence of 13 nM of the oligonucleotide Cy5P, 50 nM PolXBs and the indicated concentrations of Mn^{2+} ions. After incubation for 10 min at 30 ºC, reactions were stopped by adding 10 mM EDTA. Samples were analyzed by 7 M urea-20% PAGE and visualized using a Typhoon 9410 scanner (GE Healthcare). Full length gels are presented in Figure S8.

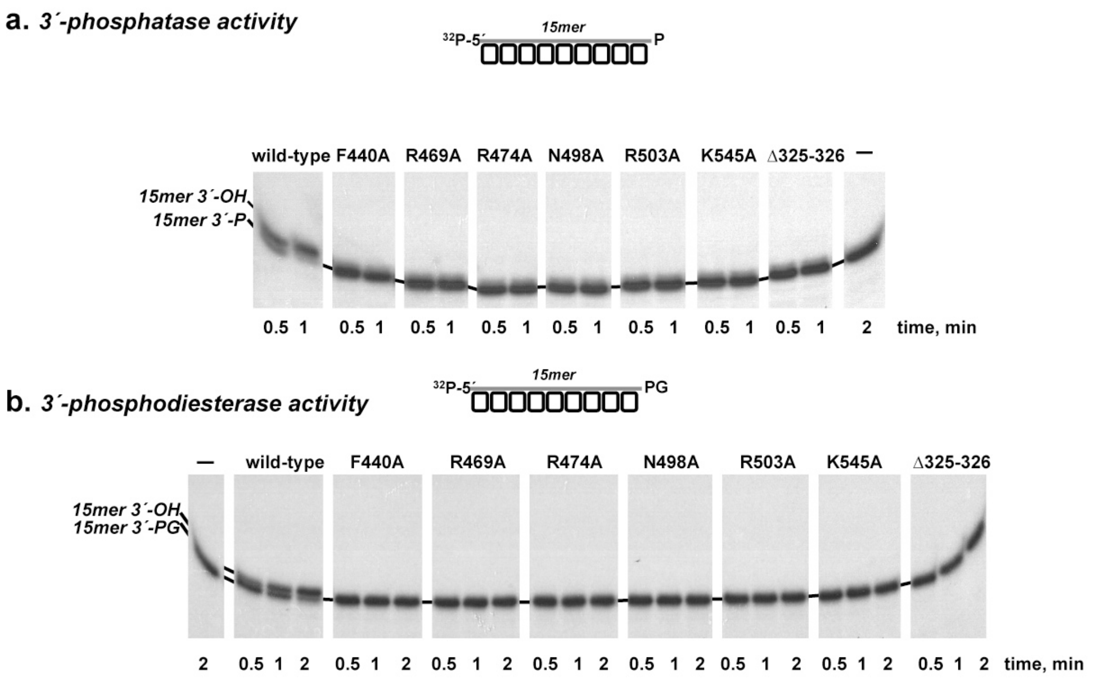

Supplementary Figure S2. Analysis of the 3´-phosphatase **(a)** and 3´-phosphodiesterase **(b)** activities of PolXBs mutants. The assay was performed as described in Materials and Methods, in the presence of 1 nM of either oligonucleotide 3´-P (a) or 3´-PG (b), and either 2.5 nM (a) or 5 nM (b) of PolXBs. After incubation for the indicated times, the reactions were stopped by adding EDTA to 10 mM. Samples were analyzed by 7 M urea-20% PAGE and further autoradiography. Full length gels are presented in Figure S9.

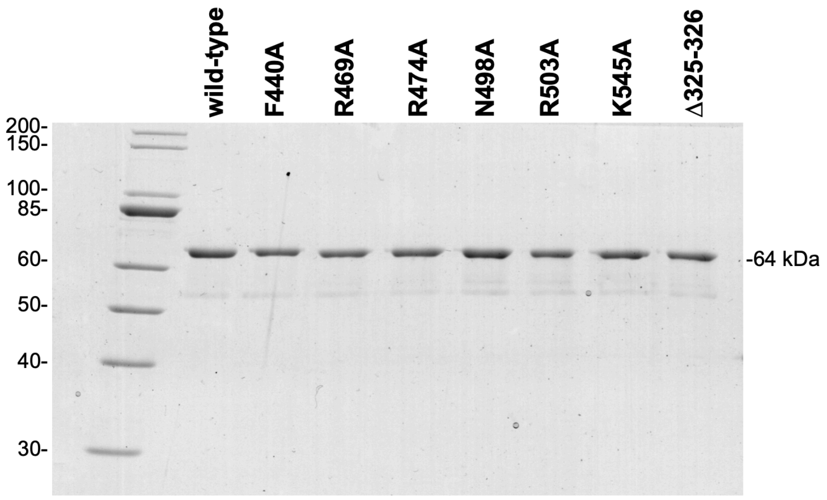

Supplementary Figure S3. Electrophoresis of purified PolXBs mutants. Aliquots (1 μg) of the purified preparations of wild-type and the indicated mutants were analyzed in 12% SDS-PAGE. Polypeptides were visualized by staining the gel with Coomassie blue dye. The positions and size (in kDa) of the marker polypeptides are indicated on the left. Full length gel is presented in Figure S10

Table S1. *Steady-state kinetic parameters of the AP-endonuclease activity of the wild-type and mutant derivatives of PolXBs*

Enzyme	k_{cat} (min^{-1})	K_D (nM)	n
**wild-type**	2.8±0.2	36±7	1.1±0.1
**F440A**	$4.2 \times 10^{-3} \pm (2 \times 10^{-4})$	24±3	1.6±0.3
**R469A**	$7.5 \times 10^{-3} \pm (3.3 \times 10^{-4})$	27±3	1.5±0.2
**R474A**	$3.4 \times 10^{-3} \pm (2.9 \times 10^{-4})$	52±9	1.2±0.1
**N498A**	$6.1 \times 10^{-3} \pm (2.4 \times 10^{-4})$	36±3	1.5±0.2
**R503A**	$1.1 \times 10^{-3} \pm (2.3 \times 10^{-4})$	27±14	1±0.3
**K545A**	$1.6 \times 10^{-2} \pm (5.8 \times 10^{-4})$	47±4	1.5±0.1
**Δ325-326**	$0.1 \pm (5.1 \times 10^{-3})$	70±5	1.4±0.1

Data are means ± standard error of at least three independent experiments
n: Hill coefficient

Table S2. *Kinetic parameters of the wild-type and mutant derivatives of PolXBs for the interaction with the AP containing ssDNA*

	**Wild-type**	**F440A**	**R469A**	**R474A**	**N498A**	**R503A**	**K545A**	**Δ325-326**
k_{a1} ($M^{-1}s^{-1}$)	3.82×10^4	2.83×10^4	1.39×10^4	5.44×10^4	2.37×10^3	3.32×10^4	4.57×10^3	6.55×10^4
k_{d1} (s^{-1})	0.0685	0.185	0.286	0.128	0.124	0.139	0.035	0.198
K_{D1} (M)	1.8×10^{-6}	6.5×10^{-6}	20.6×10^{-6}	2.4×10^{-6}	5.2×10^{-6}	4.2×10^{-6}	7.7×10^{-6}	3×10^{-6}
k_{a2} ($RU^{-1}s^{-1}$)	2.35×10^{-5}	5.11×10^{-6}	2.77×10^{-6}	2.74×10^{-5}	$1.29\ 10^{-5}$	8.95×10^{-6}	1.69×10^{-5}	4.27×10^{-5}
k_{d2} (s^{-1})	$2.25\ 10^{-3}$	2.93×10^{-3}	3.01×10^{-3}	2.92×10^{-3}	$2.93\ 10^{-3}$	2.3×10^{-3}	4.46×10^{-5}	2.79×10^{-3}
χ^2	1.01	0.47	0.85	0.54	0.25	1.6	0.25	0.56

k_a; association constant rate
k_d; dissociation constant rate
K_{D1}; apparent binding affinity (k_{d1}/k_{a1})
RU; Response Unit

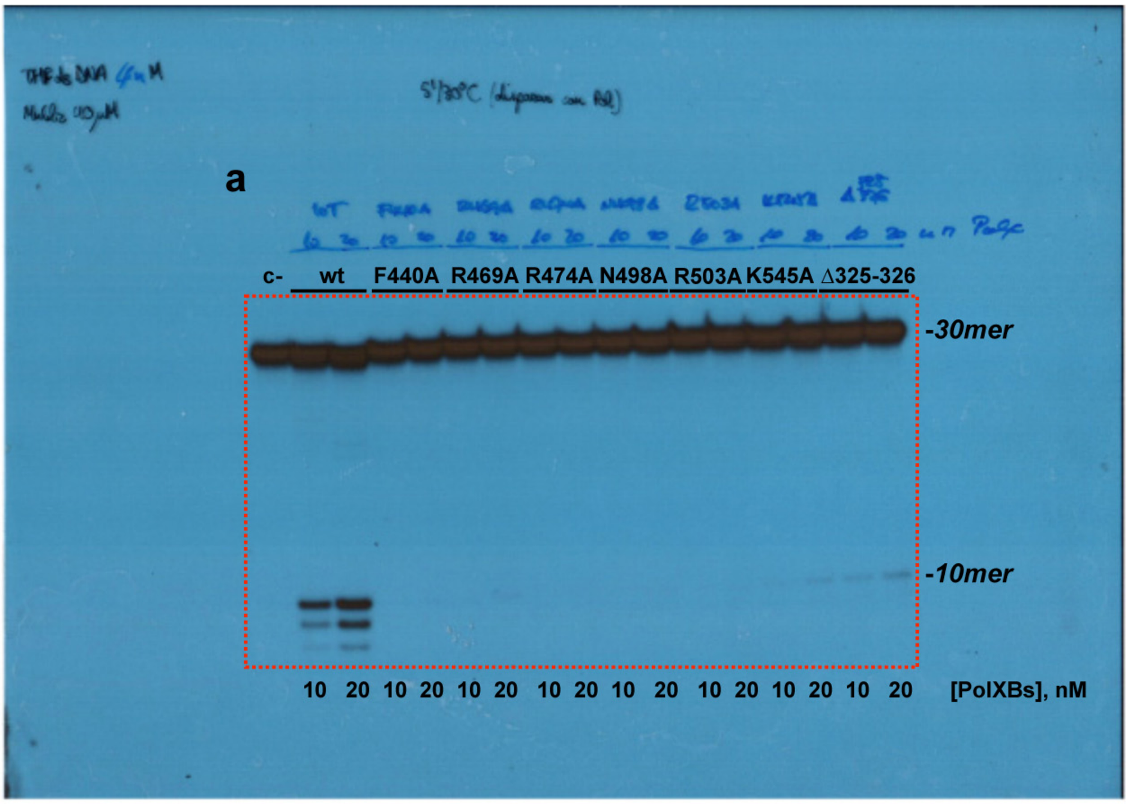

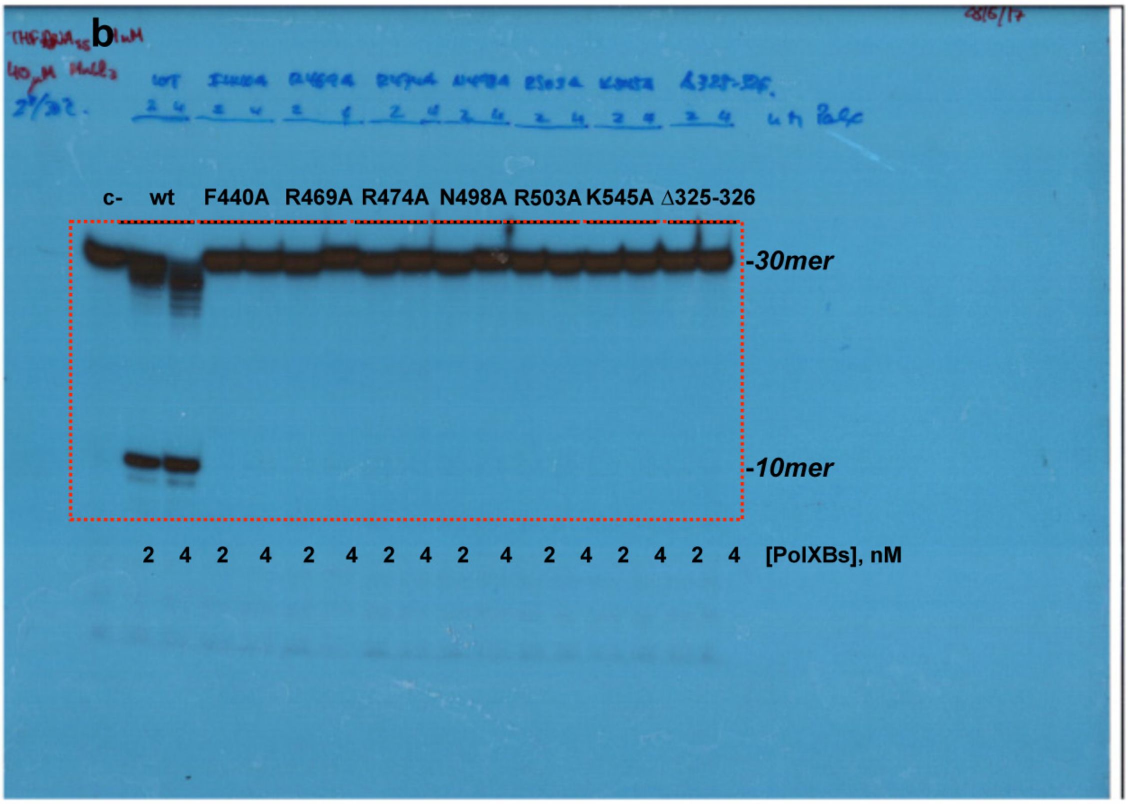

Supplementary Figure S4. Full length gels corresponding to Figure 2. Red dashed lines identify cropped regions shown in Figure 2.

A *3´-5´ exonuclease on ssDNA*

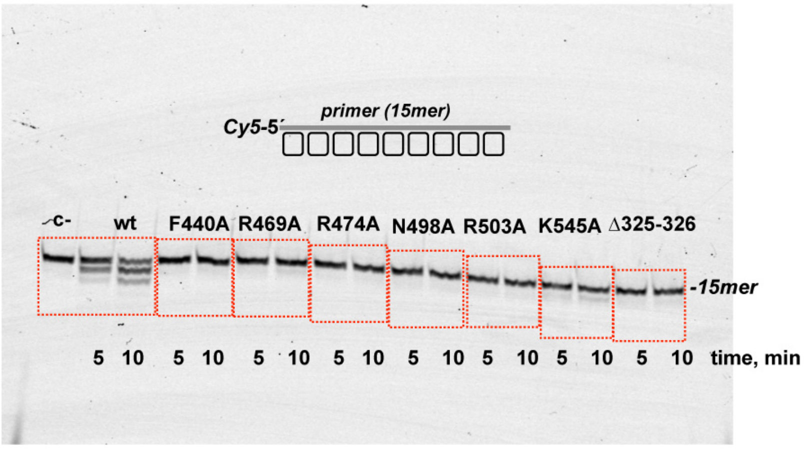

B *3´-5´ exonuclease on a gapped DNA*

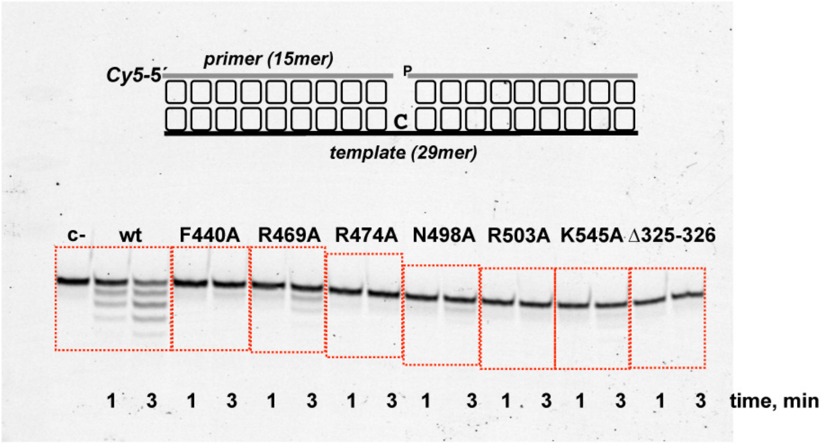

Supplementary Figure S5. Full length gels corresponding to Figure 4. Red dashed lines identify cropped regions shown in Figure 4.

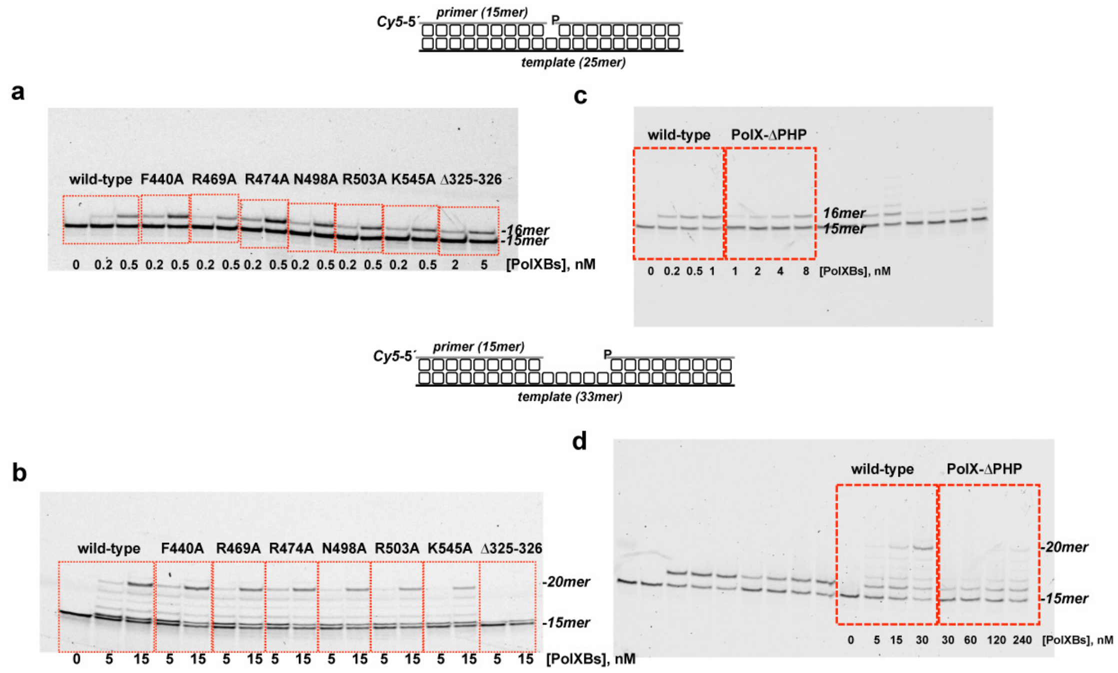

Supplementary Figure S6. Full length gels corresponding to Figure 7. Red dashed lines identify cropped regions shown in Figure 7.

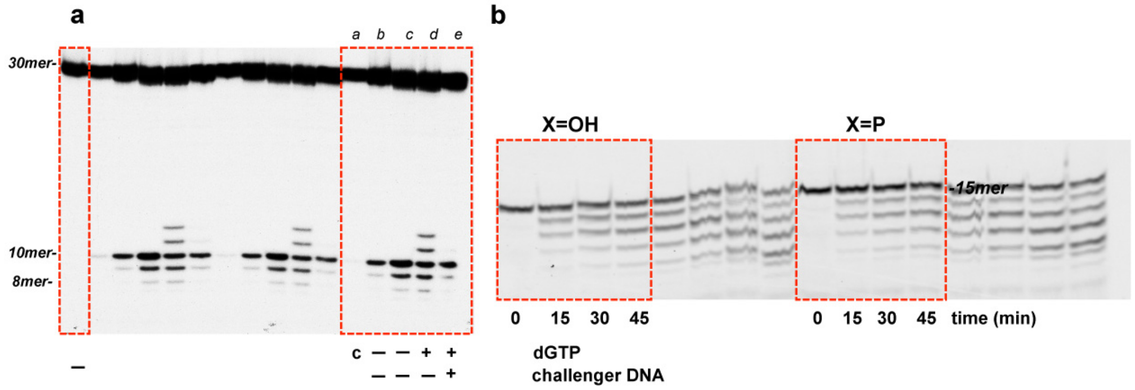

Supplementary Figure S7. Full length gels corresponding to Figure 8. Red dashed lines identify cropped regions shown in Figure 8.

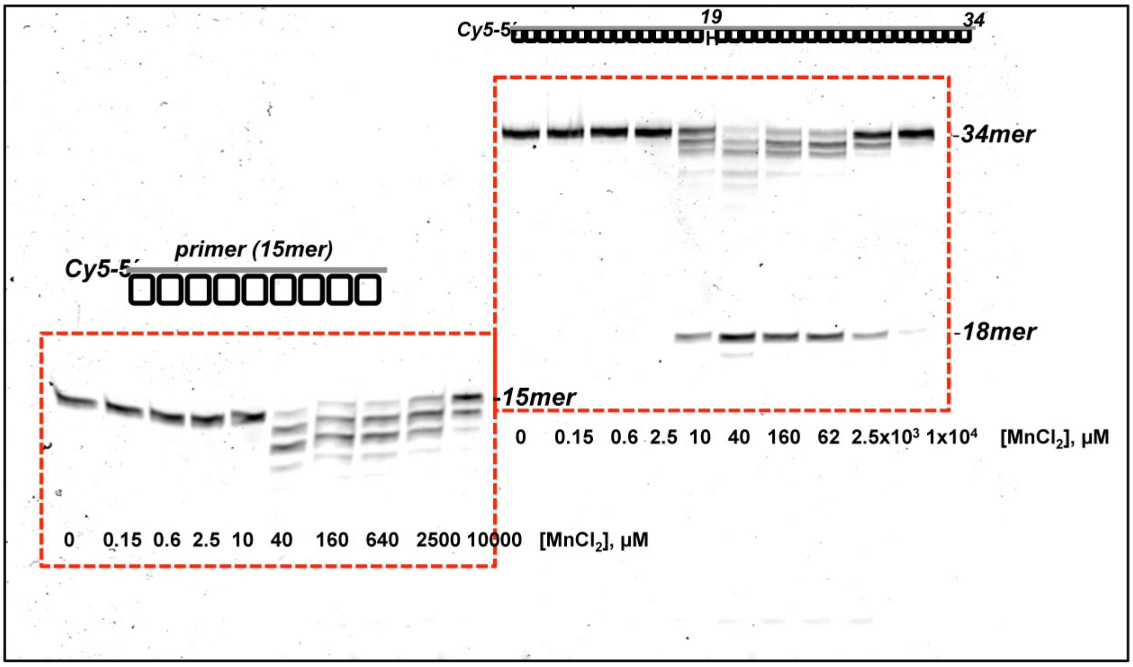

Supplementary Figure S8. Full length gel corresponding to Supplementary Figure S1. Red dashed lines identify cropped regions shown in Supplementary Figure S1.

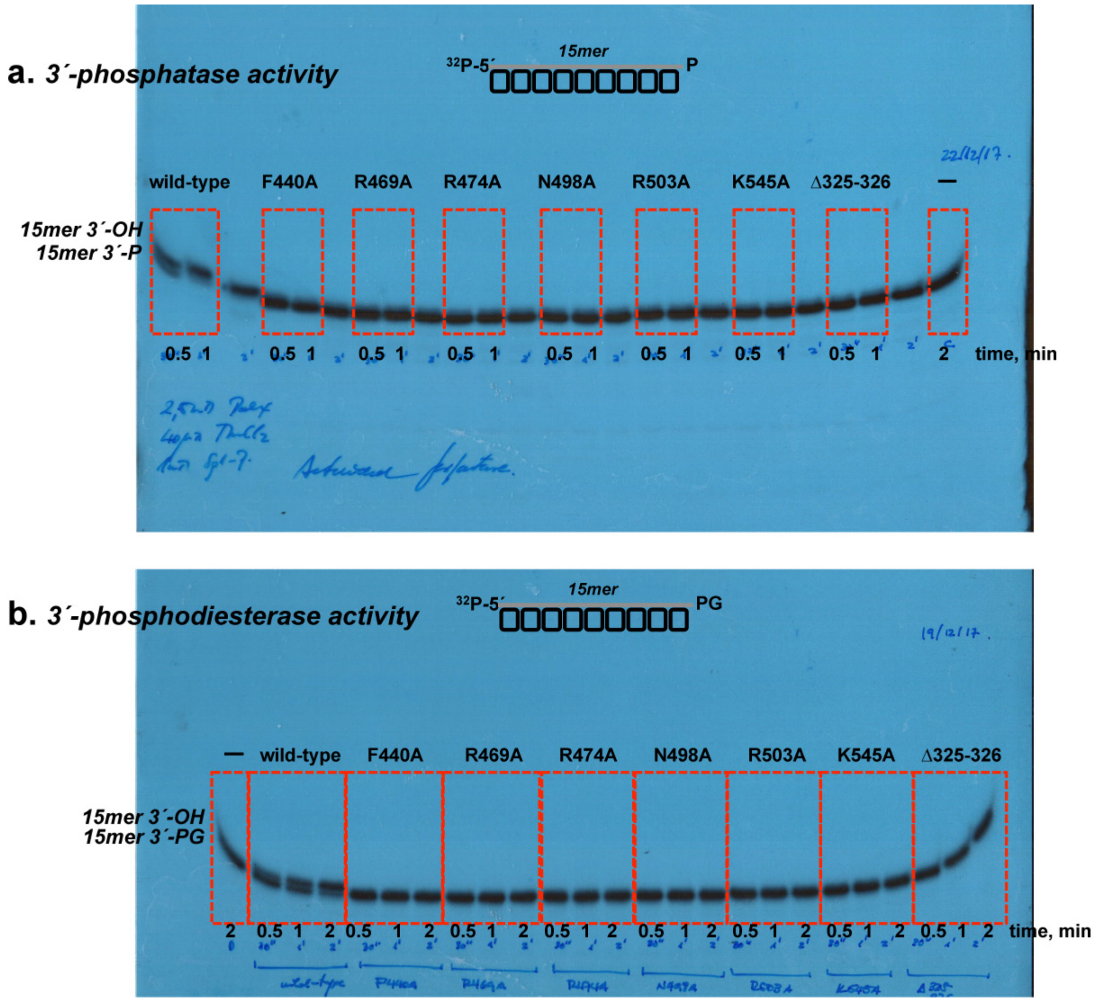

Supplementary Figure S9. Full length gels corresponding to Supplementary Figure S2. Red dashed lines identify cropped regions shown in Supplementary Figure S2.

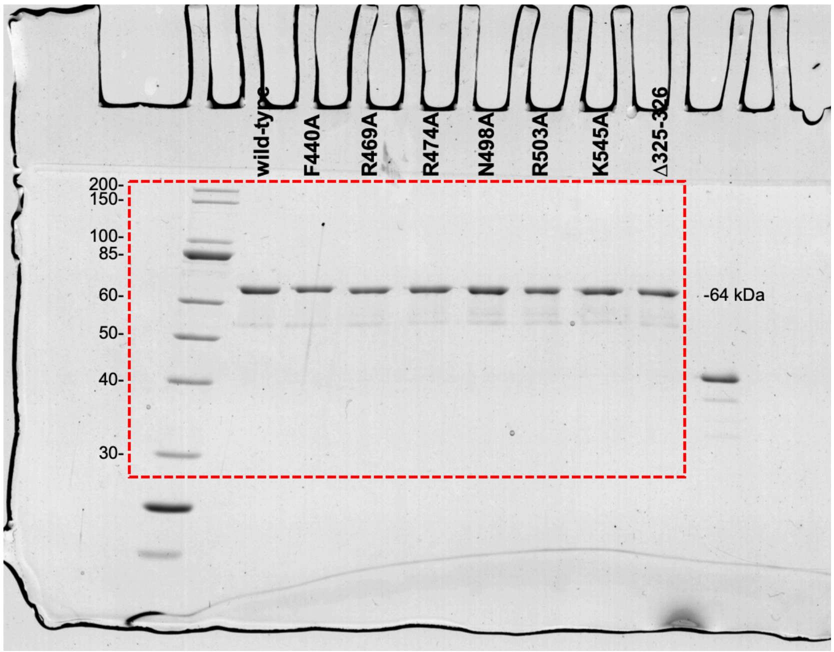

Supplementary Figure S10. Full length gel corresponding to Supplementary Figure S3. Red dashed lines identify the cropped region shown in Supplementary Figure S3.
